# Computer-assisted design model to evaluate the outcome of combined osteotomies in Legg-Calvé-Perthes disease

**DOI:** 10.3389/fped.2022.920840

**Published:** 2022-08-08

**Authors:** Hao Li, Zhiqiang Zhang, Changyou Li, Zhenpeng Liang, Zhu Liu, Hai Li, Ziming Zhang

**Affiliations:** ^1^Department of Orthopedics, Shanghai Sixth People’s Hospital, Shanghai Jiao Tong University School of Medicine, Shanghai, China; ^2^Department of Pediatric Orthopedics, Xinhua Hospital, Shanghai Jiao Tong University School of Medicine, Shanghai, China; ^3^Department of Orthopedics, National Children’s Medical Center & Children’s Hospital of Fudan University, Shanghai, China; ^4^Department of Orthopedics, Taizhou Hospital of Traditional Chinese Medicine, Linhai, China; ^5^Department of traumatic Orthopedics, Guangxi Zhuang Autonomous Region People’s Hospital, Nanning, China; ^6^Department of Pediatric Orthopedics, Shanghai Children’s Medical Center, Shanghai Jiao Tong University School of Medicine, Shanghai, China; ^7^Department of Orthopedics, Children’s Hospital Affiliated to Shanghai Jiao Tong University School of Medicine, Shanghai, China

**Keywords:** shape difference analysis, pelvic osteotomy, proximal femur osteotomy, Legg-Calvé-Perthes disease, computer-assisted design model

## Abstract

**Objective:**

The current study aims to conduct a quantitative dynamic analysis of hip morphology using a computer-assisted design (CAD) model to evaluate the combined pelvic and femoral osteotomies in the treatment of Legg-Calvé-Perthes disease (LCPD).

**Materials and methods:**

CAD models of patients with unilateral LCPD treated by combined pelvic and proximal femoral osteotomies were established based on the data of CT scan, on which morphological parameters were measured. Shape difference analysis of normal hips was adopted to locate the most apparent displacement and the main strain on the surface of the proximal femur.

**Results:**

Fifteen patients were included, and the mean age of receiving operation was 6.63 years old. There were 10 hips rated as Herring type C, and the rest were type B. Compared with the normal side, the affected hip joints have a longer distance between femoral head and acetabular sphere. The difference of coverage area of the femoral head surface and femoral head volume between the affected and normal sides was bigger compared with the preoperative model, respectively. The changes in the acetabular radius and the area of the surface were not apparent, pre-, and post-operatively. The displacement was mainly on superior and lateral superior portions of the femoral head where the stresses were concentrated.

**Conclusion:**

Combined pelvic and femoral osteotomies could effectively improve the superior and superior–posterior area of acetabulum containment with increased femoral head volume. CAD model and shape difference analysis can provide a better understanding of deformations of LCPD and more information for surgical planning and evaluation of treatment outcomes.

## Introduction

The clinical onset of Legg-Calvé-Perthes Disease (LCPD) is usually between 4 and 8 years old (1, 2). The severity, complications, and prognosis vary significantly among patients (3). There are four stages of LCPD, which are avascular necrosis (AVN), fragmentation, reconstitution, and the healed stage (4). The evolution of the disease, however, may be altered by treatment (5, 6). Containment surgery aims at increasing the coverage of the femoral head, restoring the congruency, and biomechanical environment (7, 8), and it should be performed before the advanced stage of fragmentation (9).

Several studies (6, 10–12) reported satisfying outcomes of proximal femoral varus osteotomy (PFVO) when treating Perthes diseases. However, PFVO might cause complications, such as limb length discrepancy (LLD), hip abductors insufficiency, and coxa vara. Salter innominate osteotomy (SIO) was designed to increase anterior coverage by 20–30° and lateral coverage by 10–15°, respectively (13). SIO is an effective surgical treatment that alters the natural course of LCPD in children with growth potential (12). However, some studies stated that performing SIO alone probably cannot improve the coverage and it might increase the pressure on the femoral head (14, 15). Consequently, Craig et al. (16) came up with the combination of pelvic and proximal femoral osteotomies to treat LCPD for the first time. They concluded that the combined surgery can minimize the possibility of LLD. At the same time, the pressure within the acetabulum remains normal which is vital to decrease the potential risk for AVN. Since then, several studies (14, 17–21) reported the effect of the combined surgery. Vukasinovic et al. (21) recommended that the combined surgery is valuable for the treatment of more severe cases of LCPD, especially for patients with older age (22), thus preventing the establishment of early secondary hip arthrosis. Javid et al. (14) used combined osteotomies in 20 older patients with LCPD and reported that outcomes improved with the combined osteotomies at skeletal maturity when compared to the natural history of untreated hips. However, it still remains controversial about what the proper indications are and what evaluation system should be applied in clinical practice.

The anteroposterior pelvic radiograph is the most commonly used evaluation of femoral head coverage and morphology. The two-dimensional image would provide inadequate information because the quality of the plain films depends on the projection angle and the experience of the physicians at radiographic measurement. Pioneer studies (23, 24) has already focused on the applications of 3D simulation and computer-assisted model in the diagnosis of diseases and evaluation of treatment outcomes, but it is still difficult nowadays to make the method as a routine use in clinical practice for technical and economic reasons. However, the computer-assisted design (CAD) model is definitely a promising technique in analyzing complicated anatomical structures. This study built the CAD models of the hip joints for LCPD patients, comparing with normal side, to evaluate the deformity of the affected hip and to provide enough information about the morphological changes after combined pelvic and proximal femoral osteotomies using CAD models. We hypothesize that the deformity of the hip joint is the root problem of the progressive development of the disease, and the CAD model can provide enough information to assist the doctor to determine preoperative planning.

## Materials and methods

With the institution’s Ethics Committee approval (XHEC-D-2021-007), a retrospective review was performed for 15 pediatric patients (2 women and 13 men) with the diagnosis of LCPD from March 2013 to November 2017, who met our inclusion criteria. The inclusion criteria were: (1) the LCPD patients were at the stage of fragmentation stage; (2) the LCPD patients received the combined surgeries; (3) the osteotomy site has already healed, and the implants were removed; (4) the CT scans were performed preoperatively and at the last follow-up; (5) there are no other conditions related to hip joint and the patients didn’t receive other hip operations and patients with pathological or other secondary hip diseases were excluded. The Herring classification (25) was used to evaluate the femoral head deformity. The medical records, demographic data, the age of receiving the surgery, and the duration of the implants in the body are also collected.

**FIGURE 1:**
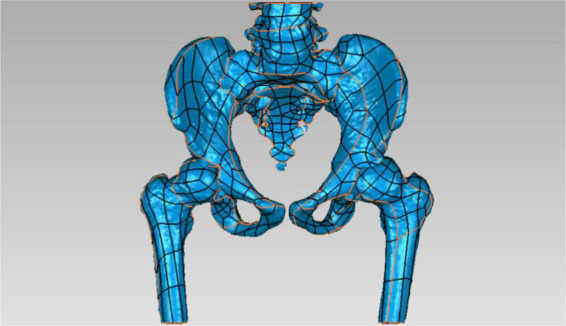
A solid model was established using the STL model in the software Geomagic Studio 11.0.

All patients underwent 64-detector row CT scans with 1.0 mm slices when the implants were removed. The raw datasets were reconstructed into a 3D STL model using MIMICS 17.0 (Materialize, Leuven, Belgium). The STL model was then imported into Geomagic Studio 11.0 (Geomagic, United States) to get a solid model (Figure 1).

The pelvic and bilateral hip joints models were imported into UG NX 10.0 (Siemens PLM Software, Charlotte, NC, United States) to measure the morphological parameters.

**FIGURE 2:**
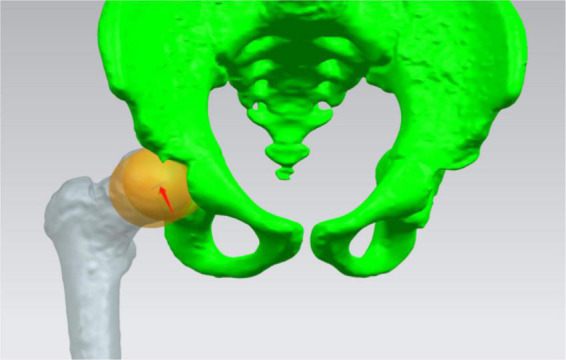
Standard spheres representing femoral head and acetabular was fitted, respectively. The distance between centers of the two spheres was measured (red arrow).

**FIGURE 3:**
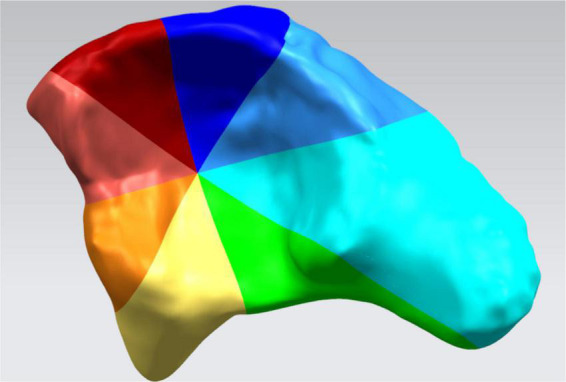
The inner surface of the acetabulum was divided into 8 sections: superior–posterior, posterior, inferior–posterior, inferior, inferior–anterior, anterior, superior–anterior, and superior.

**FIGURE 4:**
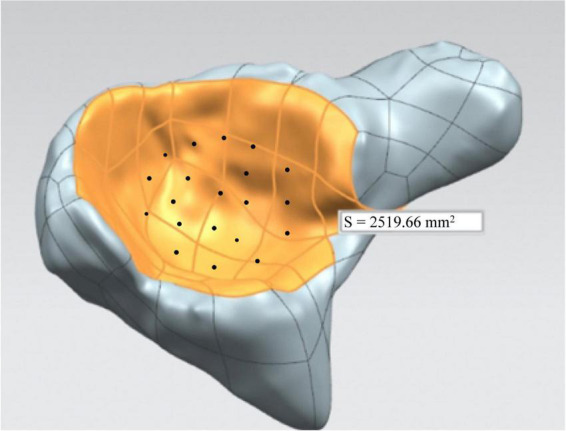
Measurements of the area of acetabular inner surface and the radius of the acetabulum.

(1) Sphere fitting:
  Standard spheres representing the femoral head and acetabular was fitted, respectively. The distance between spheres centers (SC) was measured (Figure 2), and the difference between SC (D_*SC*_) of both sides was calculated preoperatively and postoperatively according to the following calculation:

Preop. (or Postop.) D_*SC*_ = SC of the affected side - SC of the healthy side
  The difference between the preop. DSC and postop. DSC represented the imbalance of concentric structure of the affected hip joint compared to the one of the healthy side.
(2) Measurements of acetabular coverage:
  The acetabulum was divided into 8 sections to measure acetabular coverage (AC) (Figure 3) which was defined as the overlap of the best-fit femoral head sphere and corresponding section. The difference between AC (D_*AC*_) of both sides was calculated preoperatively and postoperatively according to the following calculation:

Preop. (or Postop.) D_*AC*_ = AC of the healthy side - AC of the affected side
  The effects of the operation on the acetabular coverage of affected side were assessed by the difference between preop. DAC and postop. DAC.
(3) Evaluation of defects of the femoral head:
  The models of the affected and the contralateral joints were imported into Magics 21 (Materialize, Leuven, Belgium). With the volume of the femoral head calculated, defects of the femoral head (D_*FH*_) on the affected side could be measured.
  Preop. (or Postop.) D_*FH*_ = Volume of the healthy side - Volume of the affected side
  The postop. DFH was compared with the preop. DFH. The difference between the two parameters was used to evaluate the change of femoral head volume of the affected side.
(4) Evaluation of the acetabular morphology:
  The acetabular model and the inner surface area of the acetabulum were processed in CAD software UG NX (Unigraphics Solutions, version 10.0). Twenty points on the inner surface were chosen randomly. The local acetabular radius of each point was acquired in the software. The average value of these measurements was defined as the radius of the acetabulum (Figure 4).

**FIGURE 5:**
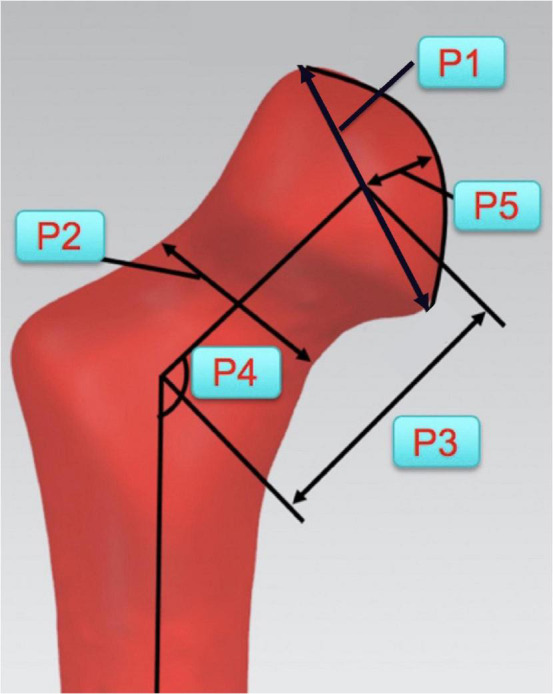
Key morphological parameters of proximal femur for establishments of average shape model. (P1: Diameter of the femoral head; P2: Diameter of the femoral neck; P3: Length of the femoral neck; P4: Angle between the axes of femoral head and shaft; and P5: Height of the femoral head).

(5) Shape difference analysis:
  On bilateral proximal femoral models of all LCPD patients, some key morphological parameters were acquired (Figure 5). The mean value of the parameters was calculated to establish an average shape model, such as a “standard” model using the data of unaffected joint. To control the difference introduced by age, all cases were divided into 2 groups according to the age of the patients (≥ 6 years old; < 6 years old). Respectively, overlap the average models of both sides and measure the distance between corresponding points on two models to estimate the shape difference.

Normality of the data distribution was tested based on the Shapiro–Wilks test. The statistical analysis was carried out with paired *t*-tests (SPSS version 19.0; SPSS, Chicago, IL, United States), comparing the preoperative parameters and the ones obtained after the removal of internal fixations. The statistical significance was set at *p* < 0.05. The demographic data were analyzed using Microsoft Excel 16.10 (Microsoft, Washington, United States).

## Results

The demographic characteristics were summarized in Tables 1, 2. The results of measurements performed on 3D CAD model demonstrated the detailed changes of hip joint (Tables 3, 4).

**TABLE 1:** The demographic data of the Legg-Calvé-Perthes disease (LCPD) patients.

Number	Sex	Side	Age at diagnosis (years)	Age at intervention (years)	Herring classification	Follow-up time (months)
1	Male	Left	6.3	6.6	C	10
2	Male	Left	6.5	7.5	B	8
3	Female	Left	6.0	8.2	B	6
4	Male	Left	5.5	6.3	B	7
5	Male	Left	8.5	9.0	B	17
6	Male	Left	7.2	8.6	C	12
7	Female	Left	5.0	5.3	B	12
8	Male	Right	5.5	6.0	C	7
9	Male	Right	5.5	6.0	C	7
10	Male	Right	4.6	5.5	C	7
11	Male	Left	4.5	5.5	C	7
12	Male	Right	5.0	5.3	C	8
13	Male	Left	7.5	8.2	C	13
14	Male	Right	4.0	7.0	C	10
15	Male	Left	3.5	4.5	C	8

**TABLE 2:** Summary of demographic data of the Legg-Calvé-Perthes disease (LCPD) patients.

Variables	Results
Male: female (%)	13 (86.7%): 2 (13.3%)
Left: right (%)	10 (66.7%): 5 (33.3%)
Average age on initial consultation (years)	5.67
Average age of receiving surgery(years)	6.63
Follow-up time (month)	9.3
**Herring classification**	
Type B	5 (33.3%)
Type C	10 (66.7%)

**TABLE 3:** Preoperative differences of parameters on computer-assisted design (CAD) models of the hip joint.

	SC (mm)	AC (mm^2^)	FH (mm^3^)
Normal side	2.23 ± 0.10	2226 ± 273.5	16412 ± 2920
Affected side	7.18 ± 6.21	2150 ± 243.7	15632 ± 2920
*p* values	0.005*	0.116	0.004*

Paired t-tests were used to compare the preoperative differences of parameters between normal and affected side. SC, sphere centers; AC, acetabular coverage; FH, femoral head; *Statistical significance.

**TABLE 4:** Changes of parameters on computer-assisted design (CAD) models of the hip joint.

	D_*SC*_ (mm)	D_*AC*_ (mm^2^)	D_*FH*_ (mm^3^)
Preop.	3.35 ± 0.81	511.12 ± 97.28	3266.14 ± 625.74
Postop.	1.05 ± 0.56	196.35 ± 71.40	1462.19 ± 541.99
*p* values	0.056	0.000*	0.001*

Paired t-tests were used to compare the differences between pre-and post-operative parameters. D_SC_, difference between bilateral distance between femoral and acetabular sphere centers; D_AC_, difference between bilateral acetabular coverage; D_FH_, defects of femoral head; Preop., preoperative; Postop., postoperative; *Statistical significance.

**FIGURE 6:**
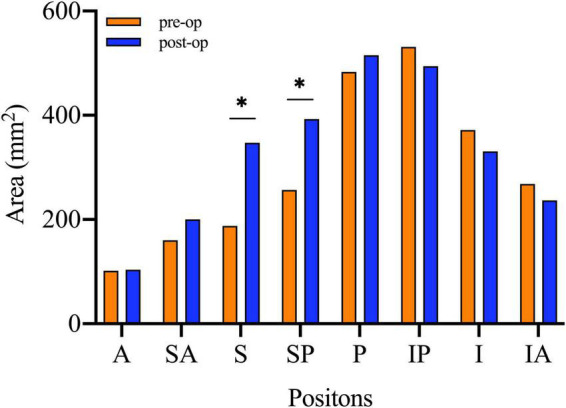
The paired *t*-tests were used to compare the area of acetabular coverage on affected side before and after the surgical intervention. A, Anterior; SA, Superior–anterior; S, Superior; SP, Superior–posterior; P, Posterior; IP, Inferior–posterior; I, inferior; IA, Inferior–anterior; Preop., Preoperative; IF, internal fixation. *Statistical significance.

**FIGURE 7:**
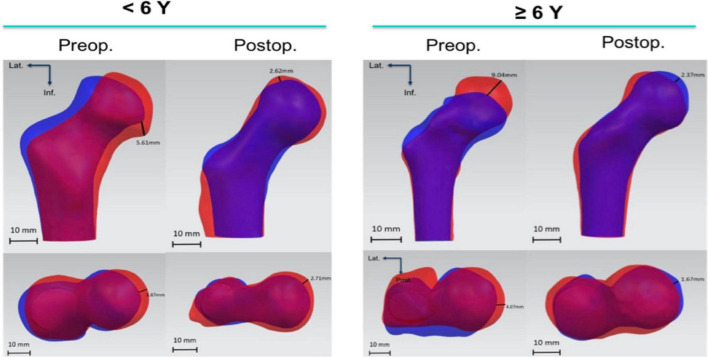
Shape difference analysis of preoperative femoral computer-assisted design (CAD) models and the ones after removal of the IF. Blue silhouette stands for affected side and red silhouette contralateral side. The illustrated distance is the maximum value of distance between corresponding points on two models.

(1) Evaluation of the concentric structure of the hip joint:
  The significant difference was observed between the normal side and the affected side of Preop. SC (*p* = 0.005). It was found that Preop. D_*SC*_ was 3.35 mm ± 0.81 mm and Postop. D_*SC*_ was 1.05 mm ± 0.56 mm, respectively. There was no significant difference (*p* = 0.056).
(2) Measurements of AC:
  There was no obvious difference in AC between the normal side and the affected side, preoperatively. D_*AC*_ decreased dramatically after the operation (Preop. D_*AC*_: 511.12 mm^2^ ± 97.28 mm^2^, Postop. D_*AC*_: 196.35 mm^2^ ± 71.40 mm^2^, *p* = 0.000). The increase in acetabular coverage mainly occurred in superior and superior–posterior sections (Figure 6).

(3) Evaluation of defects of the FH
  It was found that Preop. FH on the normal side was significantly larger than the affected side (*p* = 0.004). The Preop. D_*FH*_ was 3266.14 mm^3^ ± 625.74 mm^3^, while Postop. D_*FH*_ was 1,462.19 mm^3^ ± 541.99 mm^3^. The measurements decreased significantly at the last follow-ups (*p* = 0.001).
(4) Evaluation of acetabular morphology
  The preoperative area of the acetabular inner surface was 2,261.26 mm^2^, which was 2,519.66mm^2^ after the removal of the internal fixations. The difference was not statistically significant (*p* = 0.106). Similarly, acetabular radius did not change significantly (preoperative: 19.50 mm, postoperative: 20.65 mm, *p* = 0.291).
(5) Shape difference analysis of the femoral head
  Nine children were younger than 6 years old whereas six patients were older than six when first diagnosed. Concerning for the shape analysis, there were more shape differences between the affected femoral head (blue silhouette) and contralateral side (red silhouette) before the combined surgeries, compared with the postoperative measurements (Figure 7). Specifically, the shape analysis showed collapse and lateral extrusion of the femoral head. Additionally, as for patients older than 6 years old, the collapse of the femoral head was more obvious. After the removal of the implants, shape difference between both sides of the femoral heads became less obvious. At the final follow-up, femoral head defects of the affected side are mainly located in superior and medial parts. Compared with the preoperative average model, the femoral head had obviously shifted medially.

## Discussion

It is still controversial about the treatment options for LCPD. Although it is a self-limited condition, in some patients, LCPD can cause severe joint deformities. It is one of the most important predictive factors for the risk of developing osteoarthritis in the long term. The goal of LCPD treatment is to prevent or minimize the deformity (26). However, there is no consensus treatment protocol yet.

The age of initial onset and receiving operation is one of the factors which can alter the outcome of the treatment and the prognosis of the condition. Several studies (3, 27) found that the earlier onset was closely related to a better prognosis. Rosenfeld SB (28) reviewed 172 patients with a total of 188 affected hips of LCPD who onset before 6 years of age, he found that only children between the ages of 4 and 5 years and 11 months with a B/C or C lateral pillar classification of involvement have a less favorable prognosis. All the cases in this study are Herring group B and group C hips and received the combined surgeries during the fragmentation stage with an average age of 6.63 years old. However, five patients who received the combined surgery are younger than 6 years old because age is not the only indication. The severity of the condition should be also taken into consideration based on the fact that young patients with severely deformed epiphysis have poor prognoses. Oh HS (29) also evaluated the outcomes of patients with LCPD with onset before 6 years of age who were treated with conservative methods. Patients with prolonged initial and fragmentation stages showed worse outcomes and often required more active treatment to shorten the duration of the initial and fragmentation stages. Catterall (30) recommended that surgical intervention can provide a normal mechanical environment for the reconstruction of the affected femoral head, especially for those patients with the pathologies of hips belonging to Catterall groups 2, 3, and 4 or hips with “head at risk” signs.

Several studies (31, 32) described the deformation of the proximal femur in different ways. Chan et al. (24) found that the deformation mainly occurs in the femoral head and neck instead of the greater trochanter; stress concentration in the anterosuperior portion of the femoral head was observed in their study which made the deformed head shift laterally. Standefer et al. (33) reported that the volume ratios of femoral heads affected with LCPD ranged from 43 to 96% of a perfect hemisphere (*n* = 33). In our study, based on preoperative shape difference analysis, deformation mainly occurs in the femoral head and the residual head shifted laterally in LCPD. Consequently, the crux of the procedure is to lower the intra-articular pressure, especially on an anterosuperior portion of the femoral head, and to increase lateral coverage which is beneficial for the remodeling of the head.

Usually, the femoral head is collapsed in cases that need surgical intervention, especially for children older than 6 years (34). The results of this study showed that the proximal femoral deformation always occurs in the superior and superolateral portion, which may be the stress concentrated area. Therefore, normal stress distribution may also be a basis for femoral head recovery.

It is still controversial whether the combined surgery is better than femoral or pelvic osteotomy alone. Some studies (35–38) have already reported the drawbacks of the proximal femoral osteotomy alone. Coxa vara, LLD, limping, overgrowth, and impingement of the greater trochanter are not uncommon among patients who received the operations. To solve the problem, several surgeons (14, 18, 39, 40) turned to the combined surgery and evaluated the outcomes to prove the effectiveness of this operative design.

This study established the CAD model, making three-dimensional measurements to evaluate the outcomes of combined osteotomies. The results showed that, after receiving the combined surgeries, the affected hip joint’s coverage had been improved significantly. First, according to the results, the therapeutic goal can be achieved by combined surgery. Then, volume defects decrease significantly after an average duration of 9.3 months with the implants on the site. The restoration of the volume of the femoral head in a short time should be attributed to a normal mechanical environment created by the combined surgeries. To be specific, superior and superior–posterior portion coverage increase significantly, which is exactly the expectation of SIO. The “center-to-center” distance in this study refers to the distance between the centers of acetabulum and femoral head. It has been proved that the difference is smaller after the surgical intervention, however, without statistical significance. It could not be excluded that the concentric structure had not recovered well enough. Finally, postoperative inner surface area and radius of the affected acetabulum did not change with statistical significance compared to preoperative measurements. Overall, the combined surgeries can improve the acetabular coverage. Thus, it might lower the intra-articular pressure produced by single innominate osteotomy and prevents lower limb discrepancy after a single PFVO which could help in gait improvement and balancing muscle strength during the recovery.

There are some limitations in the present study. Firstly, our study only focused on bone structures of the joint. The cartilaginous nature of the femoral head in young patients is also important for morphology evaluation. However, we compared the affected side and the healthy side of the same patient, preoperatively and postoperatively, to reduce the limitation of failure to reconstruct cartilage in children. Ligaments, tendons, and muscles around the joint also play important roles in the normal function of the hip joint. Therefore, a more realistic and practical model should be established involving all kinds of structures of the hip joint. Secondly, due to a retrospective design, the present study did not have a control group which lowers the power of the evidence. Finally, inadequate follow-ups, small sample size, contralateral standard model without age-adjusted, and lack of joint function evaluation are drawbacks of this study.

## Conclusion

(1) Deformation mainly occurs in the superior and superolateral portion of the femoral head in LCPD and the residual head protruded and shifted laterally. Inadequate coverage in this area and concentrated stress may be important factors of the deformation.
(2) Restoration of the volume defects of the femoral head in Herring type B and C cases was observed after combined surgeries.
(3) The combined surgeries can increase the superior and superior–posterior portion coverage significantly.
(4) Computer-assisted design model can be used in accurate quantitative measurements of the deformation in LCPD and surgical planning.

## Data availability statement

The raw data supporting the conclusions of this article will be made available by the authors, without undue reservation.

## Ethics statement

The studies involving human participants were reviewed and approved by Ethics Committee of Xinhua Hospital Affiliated to Shanghai Jiao Tong University School of Medicine. Written informed consent to participate in this study was provided by the participants’ legal guardian/next of kin.

## Author contributions

HaoL and ZQZ contributed to the conception and design of the study and revised the manuscript. CL, ZPL, and ZL collected the data and performed the statistical analysis. HaiL provided funding and critically reviewed the manuscript. ZMZ coordinated and supervised data collection and critically reviewed the revised manuscript. All authors contributed to the manuscript revision, read, and approved the submitted version.

## References

[1] Kealey WD Moore AJ Cook S Cosgrove AP. Deprivation, urbanisation and Perthes’ disease in Northern Ireland. *J Bone Joint surg Br.* (2000) 82:167–71. 10.1302/0301-620X.82B2.0820167 10755420

[2] Margetts BM Perry CA Taylor JF Dangerfield PH. The incidence and distribution of Legg-Calvé-Perthes’ disease in liverpool, 1982–95. *Arch Dis child.* (2001) 84:351–4. 10.1136/adc.84.4.351 11259241 PMC1718709

[3] McAndrew MP Weinstein SL. A long-term follow-up of Legg-Calvé-Perthes disease. *J Bone Joint Surg Am.* (1984) 66:860–9. 10.2106/00004623-198466060-00006 6736087

[4] Waldenström H. The definite form of the coxa plana. *Acta Radiol.* (2016) 57:e79–94. 10.1177/0284185116642923 27298484

[5] Sankar WN Lavalva SM McGuire MF Jo C Laine JC Kim HKW. Does early proximal femoral varus osteotomy shorten the duration of fragmentation in perthes disease? Lessons from a prospective multicenter cohort. *J Pediatr Orthoped.* (2020) 40:e322–8. 10.1097/BPO.0000000000001451 31524767

[6] Singh KA Shah H Joseph B Aarvold A Kim HKW. Evolution of Legg-Calvé-Perthes disease following proximal femoral varus osteotomy performed in the avascular necrosis stage:a prospective study. *J child Orthopaed.* (2020) 14:58–67. 10.1302/1863-2548.14.190153 32165982 PMC7043118

[7] Joseph B Price CT. Principles of containment treatment aimed at preventing femoral head deformation in Perthes disease. *Orthopedic Clin North Am.* (2011) 42:317–27. 10.1016/j.ocl.2011.04.001 21742143

[8] Joseph B Srinivas G Thomas R. Management of perthes disease of late onset in southern India. the evaluation of a surgical method. *J Bone Joint Surg Br.* (1996) 78:625–30. 10.1302/0301-620X.78B4.0780625 8682832

[9] Joseph B Nair NS Narasimha Rao K Mulpuri K Varghese G. Optimal timing for containment surgery for Perthes disease. *J Pediatr Orthoped.* (2003) 23:601–6. 10.1097/01241398-200309000-00006 12960622

[10] Axer A Gershuni DH Hendel D Mirovski Y. Indications for femoral osteotomy in Legg-Calvé-Perthes disease. *Clin Orthopaed Related Res.* (1980) 150:78–87. 10.1097/00003086-198007000-00016 7428247

[11] Mazloumi SM Ebrahimzadeh MH Kachooei AR. Evolution in diagnosis and treatment of Legg-Calve-Perthes disease. *Arch Bone Joint Surg.* (2014) 2:86–92. 25207324 PMC4151449

[12] Thompson GH. Salter osteotomy in Legg-Calvé-Perthes disease. *J Pediatr Orthoped.* (2011) 31(Suppl 2):S192–7. 10.1097/BPO.0b013e318223b59d 21857438

[13] Salter RB. Role of innominate osteotomy in the treatment of congenital dislocation and subluxation of the hip in the older child. *J Bone Joint Surg Am.* (1966) 48:1413–39. 10.2106/00004623-196648070-00016 5921797

[14] Javid M Wedge JH. Radiographic results of combined Salter innominate and femoral osteotomy in Legg-Calvé-Perthes disease in older children. *J Child Orthopaed.* (2009) 3:229–34. 10.1007/s11832-009-0171-z 19387716 PMC2686812

[15] Castañeda P Vidal-Ruiz C Méndez A Salazar DP Torres A. How Often does femoroacetabular impingement occur after an innominate osteotomy for Acetabular Dysplasia? *Clin Orthopaed Related Res.* (2016) 474:1209–15. 10.1007/s11999-016-4721-7 26822844 PMC4814419

[16] Craig WAKW. Combined iliac and femoral osteotomies in Legg-Calve-Perthes syndrome. presented at the forty-first annual meeting of the american academy of orthopaedic surgeons, dallas, 17-22 january, 1974. *J Bone Joint Surg Am.* (1974) 56:1299–316. 10.2106/00004623-197456060-00030

[17] Bhuyan BK. Early outcomes of one-stage combined osteotomy in Legg-Calve’-Perthes disease. *Indian J Orthopaed.* (2016) 50:183–94. 10.4103/0019-5413.177581 27053809 PMC4800962

[18] Mosow N Vettorazzi E Breyer S Ridderbusch K Stücker R Rupprecht M. Outcome after combined pelvic and femoral osteotomies in patients with Legg-Calvé-Perthes Disease. *J Bone Joint Surg Am.* (2017) 99:207–13. 10.2106/JBJS.16.00255 28145951

[19] Elzohairy MM. Short follow-up evaluation of proximal femoral varus osteotomy for treatment of Legg-Calvé-Perthes disease. *J Orthopaed Traumatol.* (2016) 17:345–51. 10.1007/s10195-016-0412-0 27197968 PMC5071238

[20] Sarassa CA Herrera AM Carvajal J Gomez LF Lopez CA Rojas AF. Early clinical and radiological outcomes after double osteotomy in patients with late presentation Legg-Calvé-Perthes disease. *J Child Orthopaed.* (2008) 2:425–9. 10.1007/s11832-008-0132-y 19308538 PMC2656864

[21] Vukasinovic Z Slavkovic S Milickovic S Siqeca A. Combined salter innominate osteotomy with femoral shortening versus other methods of treatment for Legg-Calvé-Perthes disease. *J Pediatr Orthoped B.* (2000) 9:28–33. 10.1097/01202412-200001000-00006 10647106

[22] Lim KS Shim JS. Outcomes of combined shelf acetabuloplasty with femoral varus osteotomy in severe legg-calve-perthes (LCP) disease: advanced containment method for severe LCP disease. *Clin Orthoped Surg.* (2015) 7:497–504. 10.4055/cios.2015.7.4.497 26640634 PMC4667119

[23] Rueckert D Frangi AF Schnabel JA. Automatic construction of 3d statistical deformation models using non-rigid registration. In: Niessen WJ Viergever MA editors. *Medical Image Computing and Computer-Assisted Intervention – MICCAI 2001. MICCAI 2001. Lecture Notes in Computer Science.* (Vol. 2208), Berlin: Springer (2001). 10.1007/3-540-45468-3_10

[24] Chan EF Farnsworth CL Klisch SM Hosalkar HS Sah RL. 3-dimensional metrics of proximal femoral shape deformities in Legg-Calvé-Perthes disease and slipped capital femoral epiphysis. *J Orthopaed Res.* (2018) 36:1526–35. 10.1002/jor.23791 29087625 PMC6538305

[25] Herring JA Neustadt JB Williams JJ Early JS Browne RH. The lateral pillar classification of Legg-Calvé-Perthes disease. *J Pediatr Orthoped.* (1992) 12:143–50. 10.1097/01241398-199203000-00001 1552014

[26] Larson AN Sucato DJ Herring JA Adolfsen SE Kelly DM Martus JE A prospective multicenter study of Legg-Calvé-Perthes disease: functional and radiographic outcomes of nonoperative treatment at a mean follow-up of twenty years. *J Bone Joint Surg Am.* (2012) 94:584–92. 10.2106/JBJS.J.01073 22488614

[27] Ishida A Kuwajima SS Laredo Filho J Milani C. Salter innominate osteotomy in the treatment of severe Legg-Calvé-Perthes disease: clinical and radiographic results in 32 patients (37 hips) at skeletal maturity. *J Pediatr Orthoped.* (2004) 24:257–64. 10.1097/01241398-200405000-00004 15105719

[28] Rosenfeld SB Herring JA Chao JC. Legg-calve-perthes disease: a review of cases with onset before six years of age. *J Bone Joint Surg Am.* (2007) 89:2712–22. 10.2106/JBJS.G.00191 18056504

[29] Oh HS Sung MJ Lee YM Kim S Jung ST. Does the duration of each waldenström stage affect the final outcome of Legg-Calvé-Perthes Disease onset before 6 years of age? *Children (Basel).* (2021) 8:118. 10.3390/children8020118 33562093 PMC7916076

[30] Catterall A. Natural history, classification, and x-ray signs in Legg-Calvé-Perthes’ disease. *Acta Orthopaed Belgica.* (1980) 46:346–51. 7223380

[31] Allen BL Jr. Graphic analysis of femoral growth in young children with Perthes’ disease. *J Pediatr Orthoped.* (1997) 17:255–63. 10.1097/00004694-199703000-00021 9075105

[32] Shah HH Siddesh ND Joseph B. To what extent does remodeling of the proximal femur and the acetabulum occur between disease healing and skeletal maturity in perthes disease?: A Radiological Study. *J Pediatr Orthopaed.* (2008) 28:711–6. 10.1097/BPO.0b013e31818456dc 18812895

[33] Standefer KD Dempsey M Jo C Kim HKW. 3D MRI Quantification of Femoral Head Deformity in Legg-Calvé-Perthes Disease. *J Orthopaed Res.* (2017) 35:2051–8. 10.1002/jor.23484 27864891

[34] Kim HK. Legg-Calvé-Perthes disease. *J Am Acad. Orthopaed Surg.* (2010) 18:676–86. 10.5435/00124635-201011000-00005 21041802

[35] Chiarapattanakom P Thanacharoenpanich S Pakpianpairoj C Liupolvanish P. The remodeling of the neck-shaft angle after proximal femoral varus osteotomy for the treatment of Legg-Calve-Perthes syndrome. *J Med Assoc Thail.* (2012) 95(Suppl 10):S135–41. 23451452

[36] Green NE Beauchamp RD Griffin PP. Epiphyseal extrusion as a prognostic index in Legg-Calvé-Perthes disease. *J Bone Joint surg Am.* (1981) 63:900–5. 10.2106/00004623-198163060-00006 7240330

[37] Noonan KJ Price CT Kupiszewski SJ Pyevich M. Results of femoral varus osteotomy in children older than 9 years of age with Perthes disease. *J Pediatr Orthoped.* (2001) 21:198–204. 10.1097/01241398-200103000-00013 11242250

[38] Terjesen T Wiig O Svenningsen S. Varus femoral osteotomy improves sphericity of the femoral head in older children with severe form of Legg-Calvé-Perthes disease. *Clin Orthopaed Related Res.* (2012) 470:2394–401. 10.1007/s11999-011-2181-7 22101403 PMC3830087

[39] Pisecky L Großbötzl G Stevoska S Klotz MCM Haas C Gotterbarm T Short term radiological outcome of combined femoral and ilium osteotomy in pelvic reconstruction of the child. *Children (Basel).* (2022) 9:441. 10.3390/children9030441 35327813 PMC8946985

[40] Kamegaya M Morita M Saisu T Kakizaki J Oikawa Y Segawa Y. Single versus combined procedures for severely involved Legg-Calvé-Perthes disease. *J Pediatr Orthoped.* (2018) 38:312–9. 10.1097/BPO.0000000000000840 27442215

